# Comparison of the impact of propofol versus sevoflurane on early postoperative recovery in living donors after laparoscopic donor nephrectomy: a prospective randomized controlled study

**DOI:** 10.1186/s12871-020-01190-9

**Published:** 2020-10-28

**Authors:** Sangbin Han, Jaesik Park, Sang Hyun Hong, Soojin Lim, Yong Hyun Park, Min Suk Chae

**Affiliations:** 1 Department of Emergency medicine, Cheongyang Health Center County Hospital, Chungcheongnam-do, Republic of Korea; 2 Department of anesthesiology and Pain medicine, Seoul St. Mary’s Hospital, College of Medicine, The Catholic University of Korea, 222, Banpo-daero, Seocho-gu, Seoul, 06591 Republic of Korea; 3 Department of Urology, Seoul St. Mary’s Hospital, College of Medicine, The Catholic University of Korea, Seoul, Republic of Korea

**Keywords:** Propofol, Sevoflurane, Quality of recovery-40, Early ambulation, Living kidney donors

## Abstract

**Background:**

Enhancing postoperative recovery of the donor is important to encourage living kidney donation. We investigated the effects of anesthetic agents (intravenous [IV] propofol versus inhaled [IH] sevoflurane) on the quality of early recovery of healthy living kidney donors after hand-assisted laparoscopic nephrectomy (HALN) under analgesic intrathecal morphine injection.

**Methods:**

This single-center, prospective randomized controlled study enrolled 80 living donors undergoing HALN from October 2019 to June 2020 at Seoul St. Mary’s Hospital. Donors were randomly assigned to the IV propofol group or IH sevoflurane group. To measure the quality of recovery, we used the Korean version of the Quality of Recovery-40 questionnaire (QoR-40 K) on postoperative day (POD) 1, and ambulation (success rate, number of footsteps) 6–12 h after surgery and on POD 1. The pain score for the wound site, IV opioid requirement, postoperative complications including incidences of nausea/vomiting, and length of in-hospital stay were also assessed.

**Results:**

The global QoR-40 K score and all subscale scores (physical comfort, emotional state, physical independence, psychological support, and pain) were significantly higher in the IV propofol group than in the IH sevoflurane group. The numbers of footsteps at all time points were also higher in the IV propofol group. Donors in the IV propofol group had a lower incidence of nausea/vomiting, and a shorter hospitalization period.

**Conclusions:**

Total IV anesthesia with propofol led to better early postoperative recovery than that associated with IH sevoflurane.

**Trial registration:**

Clinical Research Information Service, Republic of Korea (approval number: KCT0004351) on October 18, 2019.

## Background

In patients with end-stage renal disease, kidney transplantation (KT) is beneficial in terms of quality of life, and also lowers morbidity and mortality rates relative to dialysis [1, 2]. According to the annual report of the Korean Network for Organ Sharing (2018), the requirement for KT has been increasing: 22,620 patients were on the waiting list in 2018, which was almost double that in 2011 [3]. One of the best solutions to satisfy the increasing need for KT is living kidney donation. However, the rate of KT has only increased by 3.25% [4]. Given the lack of organ donors, it is important to improve the experience of living donors and minimize the disincentives related to the procedure, such as postoperative pain/discomfort, prolonged hospital stay and time off work [5] as potential living donors’ concerns about the length of hospital stay, and time away from daily activities and work seem to affect their willingness to donate. Additionally, financial losses including direct out-of-pocket expanses along with indirect loss of wages from time off work and reduced productivity are important concerns to some potential living donors [6, 7].

The anesthetic agent is a clinically modifiable factor that can affect the quality of postoperative patient recovery in various surgical settings. Among intravenous (IV) and inhalational (IH) anesthetics, propofol and sevoflurane have been widely used as they offer safe and satisfactory anesthesia. However, these two drugs have different clinical features: IV propofol is associated with a lower incidence of postoperative nausea and vomiting (PONV) [8], a better sense of well-being [9], less postoperative pain [10, 11], but existence of pain on injection, and greater depressive effects on the cardiovascular and respiratory systems [12], while IH sevoflurane has good hemodynamic stability, organ-protective effects including a cardioprotective effect, but a high incidence of PONV [13].

Improving the quality of recovery would lead to a more favorable experience among living donors: i.e., shortened hospital stay, faster return to activities of daily living, improved satisfaction and reduced financial losses [7, 14]. The quality of recovery can be assessed based on Quality of Recovery-40 questionnaire (QoR-40) scores and postoperative ambulation. The QoR-40 is a validated instrument that is widely used to evaluate the quality of postoperative recovery. It is a self-rated questionnaire scored along the following sub-dimensions: physical comfort, emotional state, physical independence, psychological support and pain [14]. The QoR-40 K, which is the Korean version of the QoR-40, has been shown to have acceptable validity, reliability and feasibility [15]. Furthermore, many studies have suggested the importance of early ambulation for preventing postoperative complications [16–20]. The distance ambulated may be an objective indicator of functional status in the recovery period, but no universally accepted instruments are currently used to assess postoperative recovery based on the level of ambulation. Therefore, we used the ambulation success rate and number of steps to evaluate the quality of functional recovery in this study.

To our knowledge, few studies have investigated the effects of anesthetics on the early postoperative recovery of healthy living donors. Therefore, this study investigated the effects of anesthetics (i.e., IV propofol and IH sevoflurane) on the quality of early recovery based on the QoR-40 K scores and ambulation outcomes of healthy living donors undergoing hand-assisted laparoscopic nephrectomy (HALN).

## Methods

### Ethical considerations

**Fig. 1:**
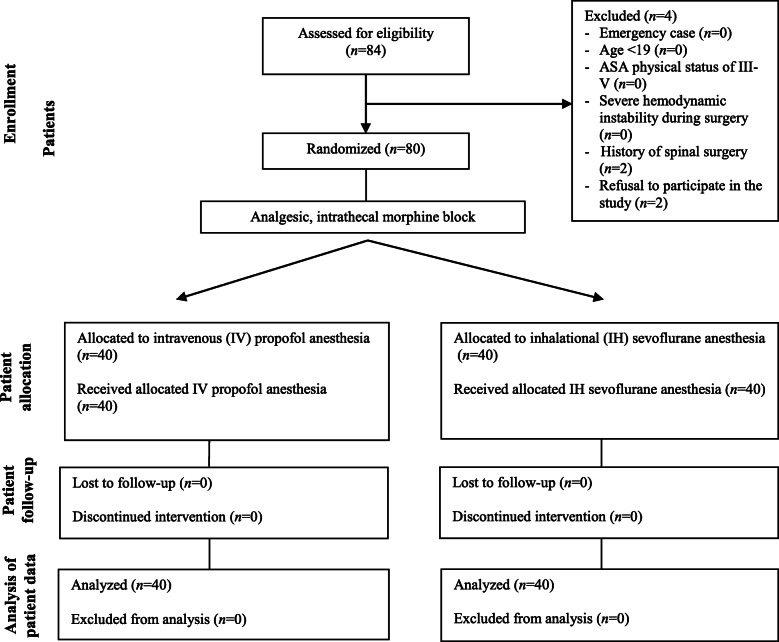
Consolidated Standards of Reporting Trials (CONSORT) flow chart

This single-center, prospective randomized controlled study was conducted at Seoul St. Mary’s Hospital. Ethical approval was obtained from the Institutional Review Board and Ethics Committee of Seoul St. Mary’s Hospital (approval number: KC19MESI0573) on October 7, 2019. The trial was performed according to the Declaration of Helsinki. The protocol was prospectively registered at a publicly accessible clinical trial database recognized by the International Committee of Medical Journal Editors (Clinical Research Information Service, Republic of Korea; approval number: KCT0004351) on October 18, 2019. Written informed consent was obtained from all patients registered in the trial between October 2019 and June 2020. Our study complies with the Consolidated Standards of Reporting Trials (CONSORT) guidelines (CONSORT Checklist); a CONSORT flow chart is presented in Fig. 1 and a summary of the study protocol is presented in Supplemental File 1.

### Study population

Adult donors (aged≥19 years) with an American Society of Anesthesiologists physical status (ASA-PS) I or II, who were suitable for kidney donation according to the clinical practice guidelines [21] and were undergoing elective HALN at our hospital, were recruited into the study. We excluded patients who refused to participate or met the following exclusion criteria: emergency case, age < 19 years, ASA-PS III or IV, intraoperative hemodynamic instability (massive hemorrhage, requirement for fluid resuscitation with colloid solution, blood product transfusion and/or infusion of strong inotropic drugs), or not appropriate for intrathecal intervention (bleeding diathesis, neurological dysfunction, history of lumbar spine surgery, recent systemic or local infection or drug allergy).

Among the 84 living donors registered in this trial, four were excluded based on the exclusion criteria: two had a history of spinal surgery and two refused to participate. Consequently, 80 living donors were included in the final analysis.

### Randomization

Living donors were randomly classified into two groups: an IV propofol group (*n* = 40) and an IH sevoflurane group (*n* = 40). We used sealed opaque envelopes to randomly assign the living donors to the groups. The envelopes were divided into groups of 10 and each group contained equal numbers of IV propofol and IH sevoflurane group allocations. A colleague not otherwise involved in this study randomly shuffled and stored the envelopes. When a participating donor entered the preoperative holding area, the uppermost envelope was opened by the attending anesthesiologist who was not a member of the investigational team and the patient was provided the anesthetic management described therein.

The attending anesthesiologist and nurses were aware of the group allocations, but were not involved in patient care after surgery. To prevent their further involvement, nurses from the postanesthetic care unit (PACU) were supervised by a member of the research team who was blinded to the group allocation. The patients, surgical team, physicians, PACU and ward nurses, and all researchers were blinded to the group allocation.

### Surgery and anesthesia

HALN, which was comprehensively described in a previous article [22], was performed in all of the patients in both groups by one experienced urological surgeon (Y.H.P). Patients were provided balanced anesthesia by the experienced attending anesthesiologist. Induction of anesthesia was achieved using 1–2 mg kg^− 1^ propofol (Fresenius Kabi, Bad Homburg, Germany) and 0.6 mg kg^− 1^ rocuronium (Merck Sharp & Dohme Corp., Kenilworth, NJ, USA). Anesthesia in the IV propofol group was maintained by infusing propofol and remifentanil (Hanlim Pharm. Co., Ltd., Seoul, Republic of Korea) according to the effect-site concentration using a target-controlled infusion pump (Orchestra® Workstation; Fresenius Kabi). Schneider’s and Minto’s pharmacokinetic models were used for propofol and remifentanil, respectively. Anesthesia in the IH sevoflurane group was maintained using sevoflurane (Hana Pharm.) combined with medical air/oxygen. In both groups, anesthetic agents were titrated to maintain the bispectral index (BIS) at 40–60. Neuromuscular blockade was maintained by additional bolus injection of rocuronium. The timing and dosage of injection were determined by the attending anesthesiologist. After the surgical procedure, neuromuscular blockade was reversed with 4 mg kg^− 1^ sugammadex (MSD Korea Ltd., Seoul, Republic of Korea) in both groups.

### Pain management

All participants received intrathecal morphine (ITM) injection and intravenous patient-controlled analgesia (IV-PCA) for postoperative analgesia. Informed consent for ITM was acquired on the day before the surgery. The ITM injection was administered before the induction of general anesthesia without any sedative. The intrathecal space was approached through the L3–4 interspace. Once free flow of cerebrospinal fluid had been observed, a single bolus of 0.2 mg (0.2 ml) morphine sulfate (BCWorld Pharm. Co., Ltd., Seoul, Republic of Korea) mixed with 0.9% saline (1 ml) to a total volume of 1.2 ml was injected slowly.

All living donors were provided with the IV-PCA device (AutoMed 3200; Ace Medical, Seoul, Republic of Korea) containing 1000 μg of fentanyl (Dai Han Pharm.) and 0.3 mg of ramosetron (Boryung Co., Ltd., Seoul, Republic of Korea) in a total volume of 100 ml. No other local anesthetic or opioid was added to the solution. The IV-PCA device was programmed as follows: no basal infusion, 1 ml bolus injection, and a lockout time of 10 min. If the numerical rating scale (NRS) pain score was ≥7 despite ITM and IV-PCA, a rescue IV opioid was administered on approval by the attending physician in the PACU or ward.

### Quality of early postoperative recovery outcomes

The quality of early postoperative recovery was evaluated using the QoR-40 K questionnaire, which consists of the following five subscales: physical comfort (12 items), emotional state (9 items), physical independence (5 items), psychological support (7 items), and pain (7 items). All items are rated on a 5-point Likert scale, where scores range from 1 (“none of the time”) to 5 (“all of the time”) for positive questions; the anchor points are reversed for negative questions. The total score can range from 40 to 200 and is calculated by summing the scores for all items. Better-quality recovery corresponds to a higher score [14]. In this study, we compared the global and all sub-dimensional scores of QoR-40 K between IV propofol and IH sevoflurane groups. Donors were asked to complete the QoR-40 K questionnaire on postoperative day (POD) 1.

We assessed functional recovery using the objective measurements of ambulation success rate and number of steps. Donors were advised to attempt sitting, standing and walking only after at least 6 h postoperatively, and only under the guidance of an attending physician. Ambulation was assessed at 6–12 h after surgery and on POD 1, at least 24 h after surgery. Successful ambulation was defined as walking more than 10 steps without any adverse event (nausea, vomiting, or pain) or physical support from the attending physician. Ambulation at the former and latter time points was classed as successful early and late ambulation, respectively. The number of steps was counted using the EI-AN900 activity tracker (Samsung Electronics, Suwon, Republic of Korea). We compared the rate of successful ambulation at early and late postoperative time points between IV propofol and IH sevoflurane groups. The numbers of steps during early and late ambulation and the total footsteps were also compared between the two groups.

### Postoperative complications

An NRS was used to evaluate the intensity of postoperative pain at the wound site. Pain severity was measured at 6 h and 24 h after surgery, and during every nursing shift as a part of standard patient care. For each measurement, donors were asked to report the intensity of pain at rest and while coughing. We collected all pain scores during the initial 24 h after surgery, and the highest NRS scores at rest and during coughing were analyzed. Total IV-PCA use and number of rescue IV opioids used during the first 24 h after surgery were also documented.

Other complications that occurred on POD 1 were recorded, including nausea/vomiting, headache, shivering, respiratory depression and pruritus. Adverse events related to the surgery were graded using the Clavien–Dindo classification, which is used to evaluate the severity of postoperative complications after many surgeries [23]. The length of hospital stay after surgery was compared between donors in the IV propofol and IH sevoflurane groups.

### Clinical variables

Preoperative findings included demographic and laboratory variables. Intraoperative findings included hemodynamic variables and total surgical duration. Laboratory variables were measured on POD 1.

### Statistical analysis

The required sample size was determined based on an unpublished retrospective pilot study conducted at Seoul St. Mary’s Hospital including 20 patients. The parameter used for the calculation of effective size was global QoR-40 K score. The number of patients needed in each group for a statistical power of 0.8 at a significance level of 5% was 36, when the standard deviation (SD) and the mean difference between groups were 30 and 20, respectively. We enrolled 40 subjects in each group assuming a dropout rate of 10%.

We used the Shapiro–Wilk test to verify the normality of the data distribution. Normally distributed data were compared using the unpaired *t*-test, while non-normally distributed data were analyzed using the Mann–Whitney *U* test. Categorical data were analyzed using Pearson’s *χ*^2^ test or Fisher’s exact test, as appropriate. Data are presented as mean ± SD, median and interquartile range, or number (%), as appropriate. All tests were two-sided. To control the overall family-wise error rate, *p*-value < 0.005 was taken to indicate statistical significance of primary outcomes. In other analyses, *p*-value < 0.05 was taken to indicate statistical significance. All statistical analyses were performed using SPSS for Windows (ver. 24.0; IBM Corp., Armonk, NY, USA) and MedCalc for Windows (ver. 11.0; MedCalc Software, Ostend, Belgium).

## Results

### Pre- and intraoperative living donor characteristics

The study population consisted of 32 (40%) male and 48 (60%) female subjects, with a mean age of 47 ± 13 years and a mean body mass index (BMI) of 23.9 ± 3.4 kg/m^2^. All living donors were in a clinically acceptable condition (ASA-PS I or II) with controlled comorbidities: two donors had a history of hypertension, but no other systemic diseases were present in the study population.

The pre- and intraoperative donor characteristics were similar between the two groups (Table 1).

**Table 1:** Comparison of pre- and intraoperative clinical findings between the IV propofol and IH sevoflurane groups

Group	IV propofol	IH sevoflurane	***p***
n	40	40	
***Preoperative findings***			
Gender (male)	18 (45.0%)	14 (35.0%)	0.361
Age (years)	50 (40–58)	49 (36–56)	0.371
Height (cm)	162.5 (156.0–170.0)	166.5 (162.0–171.5)	0.167
Weight (kg)	64.0 (54.3–71.5)	64.5 (58.0–68.8)	0.795
Body mass index (kg/m2)	24.5 (21.1–26.5)	23.4 (21.6–25.5)	0.7
*ASA physical status*			> 0.999
Status 1	32 (80.0%)	32 (80.0%)	
Status 2	8 (20.0%)	8 (20.0%)	
*Comorbidity*			
Hypertension	2 (5.0%)	0 (0.0%)	0.494
*Vital sign*			
Systolic blood pressure (mmHg)	120 (111–132)	120 (111–130)	0.742
Diastolic blood pressure (mmHg)	79 (71–80)	76 (69–80)	0.13
Heart rate (beats/min)	76 (71–85)	76 (68–82)	0.623
*Laboratory variables*			
WBC count (× 109/L)	5.6 (4.8–6.7)	5.3 (4.3–6.2)	0.163
Hemoglobin (g/dL)	13.9 (12.8–15.1)	13.9 (12.6–15.1)	0.715
Platelet count (× 109/L)	244.5 (213.5–282.3)	234.0 (215.5–280.8)	0.832
Creatinine (mg/dL)	0.77 (0.66–0.86)	0.75 (0.66–0.94)	0.647
Albumin (g/dL)	4.5 (4.3–4.7)	4.4 (4.3–4.6)	0.603
Sodium (mEq/L)	142 (141–144)	142 (141–143)	0.476
Potassium (mEq/L)	4.2 (4.0–4.4)	4.2 (4.1–4.3)	0.658
Chloride (mEq/L)	105 (103–106)	104 (103–106)	0.733
International normalized ratio	0.98 (0.95–1.02)	0.98 (0.96–1.04)	0.612
aPTT (sec)	27.2 (26.3–28.2)	26.9 (26.0–28.2)	0.56
***Intraoperative findings***			
Total surgical duration (min)	138 (125–154)	145 (126–160)	0.289
*Side of procured kidney*			0.531
Left	33 (82.5%)	35 (87.5%)	
Right	7 (17.5%)	5 (12.5%)	
*Average of vital signs*^a^			
Systolic blood pressure (mmHg)	118 (106–124)	114 (108–123)	0.476
Diastolic blood pressure (mmHg)	76 (71–83)	74 (67–79)	0.167
Heart rate (beats/min)	66 (61–74)	66 (61–76)	0.836
Hypotension event^b^	0 (0.0%)	2 (5.0%)	0.494
Total remifentanil infusion (mg)	0.5 (0.4–0.7)	0.5 (0.3–0.7)	0.154
*Total amount (mL) of*			
Fluid input	500 (400–765)	576 (400–750)	0.379
Urine output	185 (100–200)	200 (100–300)	0.115
Hemorrhage	50 (50–100)	100 (50–100)	0.229

***Abbreviations*:** *IV* Intravenous, *IH* Inhalational, *aPTT* Activated partial thrombin time

Average of vital signs^a^ were mean of values measured at three time points during surgery: immediately after induction of anesthesia, at the onset of renal arterial clamping and at the end of surgical procedure

Hypotension event^b^ was defined as systolic blood pressure < 90 mmHg over 5 min

NOTE: Values are expressed as mean median (interquartile) and number (proportion)

### QoR-40 K scores and ambulation

The global and subscale scores (i.e., physical comfort, emotional state, psychological support, physical independence, and pain) were significantly higher in the IV propofol group than in the IH sevoflurane group (Table 2). Specifically, the global QoR-40 K score was 169 (162–179) in the IV propofol group and 142 (131–154) in the IH sevoflurane group. Sub-dimension scores in the IV propofol group were 51 (47–54) for physical comfort, 41 (38–43) for emotional state, 32 (29–35) for psychological support, 17 (13–20) for physical independence and 31 (28–33) for pain while these scores in the IH sevoflurane group were 44 (38–47), 36 (32–38), 28 (25–30), 10 (8–13) and 27 (24–29), respectively.

**Table 2:** Comparison of scores in the QoR-40 K questionnaire on POD 1 between the IV propofol and IH sevoflurane groups

Group	IV propofol	IH sevoflurane	***p***
n	40	40	
Global QoR-40 K score (point)	169 (162–179)	142 (131–154)	< 0.001
*Sub-dimension score (point)*			
Physical comfort	51 (47–54)	44 (38–47)	< 0.001
Emotional state	41 (38–43)	36 (32–38)	< 0.001
Psychological support	32 (29–35)	28 (25–30)	< 0.001
Physical independence	17 (13–20)	10 (8–13)	< 0.001
Pain	31 (28–33)	27 (24–29)	< 0.001

NOTE: Values are expressed as median and interquartile

***Abbreviations*:** *IV* Intravenous, *IH* Inhalational, *QoR-40 K* Quality of Recovery-40 questionnaire, *POD* Postoperative day

The success rate of early ambulation was marginally higher in the IV propofol group (40 [100%] in the IV propofol group vs. 35 [87.5%] in the IH sevoflurane group; *p* = 0.055); however, all of the donors could ambulate on POD 1 (Table 3). The numbers of steps during the early and late postoperative periods, and the total steps on POD 1, were significantly higher in the IV propofol group than in the IH sevoflurane group. Specifically, the numbers of steps in the IV propofol group were 364 (137–516) for early ambulation, 4086 (1659–4533) for late ambulation and the total number of steps was 4449 (2179–5144), while these numbers in the IH sevoflurane group were 111 (22–398), 1730 (571–3253) and 1970 (639–3649), respectively.

**Table 3:** Comparison of postoperative ambulation between the IV propofol and IH sevoflurane groups

Group	IV propofol	IH sevoflurane	***p***
n	40	40	
Successful ambulation			
Early ambulation	40 (100.0%)	35 (87.5%)	0.055
Late ambulation	40 (100.0%)	40 (100.0%)	–
Ambulation (foot-steps)			
Total ambulation	4449 (2179–5144)	1970 (639–3649)	0.001
Early ambulation	364 (137–516)	111 (22–398)	0.004
Late ambulation	4086 (1659–4533)	1730 (571–3253)	0.001

Total ambulation was defined as sum of early and late ambulation

Early ambulation was defined as steps on the day after surgery

Late ambulation was defined as steps on postoperative day 1

NOTE: Values are expressed as number (proportion) and median (interquartile)

***Abbreviations*:** *IV* Intravenous, *IH* Inhalational

### Clinical and laboratory variables during the initial 24 h postoperatively

Nausea and vomiting was the only clinical variable that differed significantly between the two groups (Table 4). Donors in the IV propofol group had a lower incidence of nausea and vomiting than those in the IH sevoflurane group. Pain at the wound site, total IV-PCA use, rescue IV opioid use, and other clinical variables (headache, shivering, and pruritus) were similar between the groups. There were no cases of post-dural puncture headache or respiratory depression.

**Table 4:** Comparison of clinical variables during 24 h postoperatively between the IV propofol and IH sevoflurane groups

Group	IV propofol	IH sevoflurane	***p***
n	40	40	
Peak NRS score on wound site			
At rest			0.606
Mild pain (0 to 3 points)	31 (77.5%)	29 (72.5%)	
Moderate pain (4 to 6 points)	9 (22.5%)	11 (27.5%)	
Severe pain (7 to 10 points)	0 (0.0%)	0 (0.0%)	
At cough			0.612
Mild pain (0 to 3 points)	8 (20.0%)	5 (12.5%)	
Moderate pain (4 to 6 points)	18 (45.0%)	18 (45.0%)	
Severe pain (7 to 10 points)	14 (35.0%)	17 (42.5%)	
Requirement of IV opioid			
Total amount of IV-PCA infusion (mL)	13.5 (9.3–23.8)	16.0 (7.0–37.3)	0.522
Rescue IV opioid	2 (5.0%)	3 (7.5%)	> 0.999
Nausea/vomiting	12 (30.0%)	26 (65.0%)	0.002
Headache	3 (7.5%)	5 (12.5%)	0.712
Shivering	5 (12.5%)	10 (25.0%)	0.152
Respiration depression	0 (0.0%)	0 (0.0%)	–
Pruritus	11 (27.5%)	13 (32.5%)	0.626

NOTE: Values are expressed as median (interquartile) and number (proportion)

***Abbreviations*:** *IV* Intravenous, *IH* Inhalational, *NRS* Numeric rating scale, *IV-PCA* Intravenous patient-controlled analgesia

No significant differences were noted in laboratory variables between the groups on POD 1 (Table 5).

**Table 5:** Comparison of laboratory variables on POD 1 between the IV propofol and IH sevoflurane groups

Group	IV propofol	IH sevoflurane	***p***
n	40	40	
WBC count (× 109/L)	9.5 (8.4–12.1)	9.8 (8.4–10.8)	0.788
Neutrophil (%)	76.8 (72.9–80.5)	77.7 (75.1–81.5)	0.351
Lymphocyte (%)	16.5 (11.7–20.2)	14.4 (11.3–17.3)	0.142
Hemoglobin (g/dL)	11.8 (10.7–12.8)	11.6 (10.9–13.1)	0.758
Platelet count (× 109/L)	201.0 (168.3–233.5)	197.0 (165.0–237.0)	0.627
Creatinine (mg/dL)	1.3 (1.1–1.5)	1.25 (1.11–1.61)	0.988
Albumin (g/dL)	3.4 (3.3–3.6)	3.3 (3.1–3.5)	0.081
Sodium (mEq/L)	139 (138–140)	138 (137–140)	0.072
Potassium (mEq/L)	3.9 (3.8–4.3)	3.9 (3.7–4.2)	0.706
Chloride (mEq/L)	104 (103–106)	104.0 (102.0–105.8)	0.402

NOTE: Median and interquartile

***Abbreviations*:** *WBC* White blood cell, *IV* Intravenous, *IH* Inhalationalm, *POD* Postoperative day

### Surgical complications and length of hospital stay

All donors were classified as Clavien–Dindo grade 1 and discharge was uneventful in all cases. The length of hospital stay after surgery was significantly shorter in the IV propofol group than in the IH sevoflurane group (3 [3, 4] days in the IV propofol group versus 4 [3–5] days in the IH sevoflurane group; *p* = 0.013).

## Discussion

The main finding of our study was that the global QoR-40 K score was significantly higher in donors receiving IV propofol than in those receiving IH sevoflurane; this tendency was also observed for the physical comfort, emotional state, physical independence, psychological support and pain subscale scores. The numbers of steps during early and late ambulation, and the total number of steps (which was taken to reflect physical capability) were also higher in the IV propofol group than in the IH sevoflurane group. Moreover, the length of hospital stay was shorter in the IV propofol group than in the IH sevoflurane group.

The better recovery outcomes (higher QoR-40 K score and physical capability) observed in the IV propofol group could be explained by differences in characteristics between the two anesthetic agents. First, propofol has anxiolytic effects and produces a general sense of well-being, or even euphoria, after general anesthesia [9, 24, 25]. The anxiolytic effect is related to potentiation of GABA_A_ receptors and inhibition of the serotonergic system, while the euphoric mood is associated with the stimulation of dopaminergic neurons in the ventral tegmental area [24, 26]. These effects of propofol on patient mood may have contributed to the higher scores on the emotional subscales of the QoR-40 K, considering that some donors have been reported to experience short-term mood changes after organ donation [27]. Second, many previous studies have demonstrated that propofol has analgesic and antinociceptive effects [24]. The analgesic effect of sevoflurane is also widely known, but which agent provides superior postoperative analgesic effects remains controversial, with equivocal results among studies [10, 11]. In the present trial, a higher pain score on the QoR-40 K, indicating less pain, was observed in the IV propofol group. However, as the pain subscale of the QoR-40 K subsumes extra-surgical pain, such as muscle pain, headache and backache, it is unclear whether the IV propofol actually provided better postoperative analgesia at the wound site. In this trial, the highest NRS pain score for the wound site was slightly, but non-significantly, lower in the IV propofol group, both during coughing and at rest. Third, propofol significantly reduces PONV compared with inhalational anesthetics [28]. The antiemetic effect of propofol is associated with inhibition of the 5-hydroxy-tryptamine-3 (5-HT) receptors in the serotonergic system, dopaminergic (D2) receptors in the chemoreceptor trigger zone, and the limbic system [24, 29]. PONV is not only covered by a separate item in the QoR-40 K questionnaire, but also affects the overall sense of physical comfort [30]. This is consistent with our findings of a significantly lower incidence of PONV and higher physical comfort scores in the IV propofol group. Fourth, sevoflurane leads to a greater decrease of bronchial mucus transport relative to propofol. Impaired bronchociliary clearance may have resulted in the retention of secretions, which can cause discomfort while breathing after surgery, as well as a higher risk of pulmonary complications [31]. Finally, a modulatory effect on surgical stress of propofol, as well as anti-inflammatory effects, have been demonstrated in previous studies [32–34]. It is well known that surgical injury triggers the systemic inflammatory response (SIR), where an excessive SIR is assumed to contribute to delayed recovery after surgery and postoperative complications [35, 36]. SIR may have played a role in the better recovery of our donors who received IV propofol.

However, some recent studies have reported no differences in the effects of propofol and sevoflurane on postoperative recovery outcomes, namely the QoR-40 scores, postoperative pain, length of PACU stay, and complications, including PONV [37–39]. Possible explanations for this discrepancy between our results and those of previous studies include different study population characteristics, surgical etiologies and analgesic regimens. The donors in our study were healthier; most had no comorbidities, except for two with controlled hypertension. Although other studies have enrolled patients with ASA-PS of I or II, they did not investigate comorbidities, and the patients were undergoing surgery due to their illness. Differences in underlying health conditions among study populations could confound comparison of postoperative recovery. In one recent study, higher level of physical activity in the pre-donation period was positively associated with the occurrence of chronic postsurgical pain, indicating that donors involved in vigorous physical activities may be more sensitive to postoperative pain or discomfort than stationary donors [40]. Additionally, pain threshold tends to be lower in healthy living donors than in patients undergoing a similar surgical procedure for health reasons, which could also have affected the results [41]. We used ITM as the analgesic in this study, which offers superior analgesia compared with IV opioid, IV-PCA, and continuous wound infusion, for example [42]. Better pain control, and subsequently reduced IV opioid consumption and PONV incidence, may facilitate ambulation, improve physical capability, and prevent severe wound pain, thus resulting in a shorter hospitalization period [43, 44].

Several limitations of this study should be discussed. First, the specific mechanisms underlying the differences in recovery were not determined. Second, we calculated the sample size required to detect group differences in the QoR-40 K scores, rather than in subscale scores or other clinical variables. Third, as this study was performed in healthy donors undergoing HALN in the setting of ITM, the results may not be generalizable to other patient populations, surgeries, or analgesic strategies. Finally, no long-term follow-up was performed.

## Conclusions

The choice of anesthetic drug may affect the quality of early postoperative recovery in healthy living donors undergoing HALN. IV propofol seems to be a better option with respect to postoperative recovery than IH sevoflurane under appropriate analgesia, such as ITM.

## Supplementary information

**Additional file 1: Supplemental file 1.** Summary of our study protocol.

## Data Availability

The datasets used and/or analyzed during this study are available from the corresponding author on reasonable request.

## Abbreviations

KT: Kidney transplantation
IV: Intravenous
IH: Inhalational
PONV: Postoperative nausea and vomiting
QoR-40: Quality of Recovery-40
QoR-40 K: The Korean version of the QoR-40
CONSORT: Consolidated Standards of Reporting Trials
HALN: Hand-assisted laparoscopic nephrectomy
ASA-PS: American Society of Anesthesiologists physical status
PACU: Post-anesthetic care unit
BIS: Bispectral index
ITM: Intrathecal morphine
IV-PCA: Intravenous patient-controlled analgesia
NRS: Numerical rating scale
POD: Postoperative day
SD: Standard deviation
BMI: Body mass index
5-HT: 5-hydroxy-tryptamine-3
IL: Interleukin
SIR: Systemic inflammatory response

## References

[1] Hart A, Smith J, Skeans M, Gustafson S, Wilk A, Castro S (2020). OPTN/SRTR 2018 annual data report: kidney. Am J Transplant.

[2] Rose C, Gill J, Gill JS (2017). Association of Kidney Transplantation with survival in patients with long dialysis exposure. Clin J Am Soc Nephrol.

[3] Korean Network for Organ Sharing (KONOS) (2019). Organ transplantation and donation of human tissue statistical yearbook 2018.

[4] Rege A, Leraas H, Vikraman D, Ravindra K, Brennan T, Miller T (2016). Could the use of an enhanced recovery protocol in laparoscopic donor nephrectomy be an incentive for live kidney donation?. Cureus..

[5] Ashcraft EE, Baillie GM, Shafizadeh SF, McEvoy JR, Mohamed HK, Lin A (2001). Further improvements in laparoscopic donor nephrectomy: decreased pain and accelerated recovery. Clin Transpl.

[6] Boulware LE, Ratner LE, Sosa JA, Tu AH, Nagula S, Simpkins CE (2002). The general Public's concerns about clinical risk in live kidney donation. Am J Transplant.

[7] Klarenbach S, Gill J, Knoll G, Caulfield T, Boudville N, Prasad G (2014). Economic consequences incurred by living kidney donors: a Canadian multi-center prospective study. Am J Transplant.

[8] Kumar G, Stendall C, Mistry R, Gurusamy K, Walker D (2014). A comparison of total intravenous anesthesia using propofol with sevoflurane or desflurane in ambulatory surgery: systematic review and meta-analysis. Anesthesia..

[9] Hofer CK, Zollinger A, Buchi S, Klaghofer R, Serafino D, Buhlmann S (2003). Patient well-being after general anesthesia: a prospective, randomized, controlled multi-Centre trial comparing intravenous and inhalation anesthesia. Br J Anaesth.

[10] Fassoulaki A, Melemeni A, Paraskeva A, Siafaka I, Sarantopoulos C (2008). Postoperative pain and analgesic requirements after anesthesia with sevoflurane, desflurane or propofol. Anesth Analg.

[11] Li M, Mei W, Wang P, Yu Y, Qian W, Zhang ZG (2012). Propofol reduces early post-operative pain after gynecological laparoscopy. Acta Anaesthesiol Scand.

[12] Fredman B, Nathanson MH, Smith I, Wang J, Klein K, White P (1995). Sevoflurane for outpatient anesthesia: a comparison with Propofol. Anesth Analg.

[13] Brioni JD, Varughese S, Ahmed R, Bein B (2017). A clinical review of inhalation anesthesia with sevoflurane: from early research to emerging topics. J Anesth.

[14] Myles PS, Weitkamp B, Jones K, Melick J, Hensen S (2000). Validity and reliability of a postoperative quality of recovery score: the QoR-40. Br J Anaesth.

[15] Lee JH, Kim D, Seo D, Son JS, Kim DC (2018). Validity and reliability of the Korean version of the quality of Recovery-40 questionnaire. Korean J Anesthesiol.

[16] Cassidy MR, Rosenkranz P, McAneny D (2014). Reducing postoperative venous thromboembolism complications with a standardized risk-stratified prophylaxis protocol and mobilization program. J Am Coll Surg.

[17] Haines KJ, Skinner EH, Berney S, Investigators AHPS (2013). Association of postoperative pulmonary complications with delayed mobilisation following major abdominal surgery: an observational cohort study. Physiotherapy..

[18] Wenger N (1978). The physiologic basis for early ambulation after myocardial infarction. Cardiovasc Clin.

[19] Parker HG, Reitman HK (1976). Changing patterns in fracture management emphasizing early motion and function. Surg Clin N Am.

[20] Lee T-G, Kang S-B, Kim D-W, Hong S, Heo SC, Park KJ (2011). Comparison of early mobilization and diet rehabilitation program with conventional care after laparoscopic colon surgery: a prospective randomized controlled trial. Dis Colon Rectum.

[21] Mandelbrot DA, Reese PP, Garg N, Thomas CP, Rodrigue JR, Schinstock C (2020). KDOQI US commentary on the 2017 KDIGO clinical practice guideline on the evaluation and Care of Living Kidney Donors. Am J Kidney Dis.

[22] Seo SI, Kim JC, Hwangbo K, Park YH, Hwang TK (2005). Comparison of hand-assisted laparoscopic and open donor nephrectomy: a single-center experience from South Korea. J Endourol.

[23] Dindo D, Demartines N, Clavien PA (2004). Classification of surgical complications: a new proposal with evaluation in a cohort of 6336 patients and results of a survey. Ann Surg.

[24] Vasileiou I, Xanthos T, Koudouna E, Perrea D, Klonaris C, Katsargyris A (2009). Propofol: a review of its non-anaesthetic effects. Eur J Pharmacol.

[25] Brechmann T, Maier C, Kaisler M, Vollert J, Schmiegel W, Pak S (2018). Propofol sedation during gastrointestinal endoscopy arouses euphoria in a large subset of patients. United European Gastroenterol J.

[26] Li KY, Xiao C, Xiong M, Delphin E, Ye JH (2008). Nanomolar propofol stimulates glutamate transmission to dopamine neurons: a possible mechanism of abuse potential?. J Pharmacol Exp Ther.

[27] Jowsey SG, Jacobs C, Gross CR, Hong BA, Messersmith EE, Gillespie BW (2014). Emotional well-being of living kidney donors: findings from the RELIVE study. Am J Transplant Off J Am Soc Transplant Am Soc Transplant Surg.

[28] Yoo YC, Bai SJ, Lee KY, Shin S, Choi EK, Lee JW (2012). Total intravenous anesthesia with propofol reduces postoperative nausea and vomiting in patients undergoing robot-assisted laparoscopic radical prostatectomy: a prospective randomized trial. Yonsei Med J.

[29] Barann M, Göthert M, Fink K, Bönisch H (1993). Inhibition by anaesthetics of 14 C-guanidinium flux through the voltage-gated sodium channel and the cation channel of the 5-HT 3 receptor of N1E-115 neuroblastoma cells. Naunyn Schmiedeberg's Arch Pharmacol.

[30] Lee WK, Kim MS, Kang SW, Kim S, Lee JR (2015). Type of anaesthesia and patient quality of recovery: a randomized trial comparing propofol-remifentanil total i.v. anaesthesia with desflurane anaesthesia. Br J Anaesth.

[31] Ledowski T, Paech MJ, Patel B, Schug SA (2006). Bronchial mucus transport velocity in patients receiving propofol and remifentanil versus sevoflurane and remifentanil anesthesia. Anesth Analg.

[32] Gilliland HE, Armstrong MA, Carabine U, McMurray TJ (1997). The choice of anesthetic maintenance technique influences the antiinflammatory cytokine response to abdominal surgery. Anesth Analg.

[33] Ke J, Zhan J, Feng X, Wu Y, Rao Y, Wang Y (2008). A comparison of the effect of total intravenous anesthesia with propofol and remifentanil and inhalational anesthesia with isoflurane on the release of pro-and anti-inflammatory cytokines in patients undergoing open cholecystectomy. Anesthesia Intensive Care.

[34] Kotani N, Hashimoto H, Sessler DI, Yasuda T, Ebina T, Muraoka M (1999). Expression of genes for proinflammatory cytokines in alveolar macrophages during propofol and isoflurane anesthesia. Anesth Analg.

[35] Crippa J, Mari GM, Miranda A, Costanzi AT, Maggioni D (2018). Surgical stress response and enhanced recovery after laparoscopic surgery - a systematic review. Chirurgia (Bucur).

[36] Watt DG, Horgan PG, McMillan DC (2015). Routine clinical markers of the magnitude of the systemic inflammatory response after elective operation: a systematic review. Surgery..

[37] Carli D, Meletti JFA, Neto NEU, Martinez G, Kim ALC, de Camargo RPS (2020). General anesthesia technique and perception of quality of postoperative recovery in women undergoing cholecystectomy: a randomized, double-blinded clinical trial. PLoS One.

[38] De Oliveira GS, Jr., Bialek J, Rodes ME, Kendall MC, McCarthy RJ. (2017). The effect of sevoflurane compared to propofol maintenance on post-surgical quality of recovery in patients undergoing an ambulatory gynecological surgery: a prospective, randomized, double-blinded, controlled, clinical trial. J Clin Anesth.

[39] Moro ET, Leme FC, Noronha BR, Saraiva GF, de Matos Leite NV, Navarro LH (2016). Quality of recovery from anesthesia of patients undergoing balanced or total intravenous general anesthesia. Prospective randomized clinical trial. J Clin Anesth.

[40] Fleishman A, Khwaja K, Schold JD, Comer CD, Morrissey P, Whiting J (2020). Pain expectancy, prevalence, severity, and patterns following donor nephrectomy: findings from the KDOC study. Am J Transplant.

[41] Cywinski JB, Parker BM, Xu M, Irefin SA (2004). A comparison of postoperative pain control in patients after right lobe donor hepatectomy and major hepatic resection for tumor. Anesth Analg.

[42] Tang JZJ, Weinberg L. A Literature Review of Intrathecal Morphine Analgesia in Patients Undergoing Major Open Hepato-Pancreatic-Biliary (HPB) Surgery. Anesthesiol Pain Med. 2019;9(6):e94441. 10.5812/aapm.94441 PMC7118737 32280615

[43] Dichtwald S, Ben-Haim M, Papismedov L, Hazan S, Cattan A, Matot I (2017). Intrathecal morphine versus intravenous opioid administration to impact postoperative analgesia in hepato-pancreatic surgery: a randomized controlled trial. J Anesth.

[44] Cheah JW, Sing DC, Hansen EN, Aleshi P, Vail TP (2018). Does intrathecal morphine in spinal anesthesia have a role in modern multimodal analgesia for primary Total joint arthroplasty?. J Arthroplast.

