# Supplementary material for: Comparison of the impact of propofol versus sevoflurane on early postoperative recovery in living donors after laparoscopic donor nephrectomy: a prospective randomized controlled study
Source: BMC Anesthesiol. 2020 Oct 28;20:273. doi: 10.1186/s12871-020-01190-9 (PMC7592560; doi:10.1186/s12871-020-01190-9)
Supplement: Supplementary file 1 — Additional file 1: Supplemental file 1. Summary of our study protocol. [file 12871_2020_1190_MOESM1_ESM.docx]

| **Supplemental file 1.** Summary of our study protocol | |
| --- | --- |
| **Study design** | Prospective randomized controlled study |
| **Funding** | No grants and financial support to declare. |
| **Trial registration** | The International Committee of Medical Journal Editors (Clinical Research Information Service, Republic of Korea; approval number: KCT0004351) on October 18, 2019 |
| **Object** | To compare the effects of anesthetic agents, intravenous propofol versus inhalational sevoflurane, on the quality of early recovery of healthy living kidney donors after hand-assisted laparoscopic nephrectomy under analgesic intrathecal morphine injection. |
| ***Enrolment*** | |
| **Assessed for eligibility** |  |
| **Physical and laboratory assessments before surgery** | |
| **Inclusion criteria** | 1) Hand-assisted laparoscopic nephrectomy for kidney transplantation  2) Elective surgery 3) ASA physical status I or II |
| **Exclusion criteria** | 1) Emergency surgery 2) Age<19 years 3) ASA physical status III - V 4) Intraoperative hemodynamic instability  5) Contraindication for intrathecal intervention 6) Refusal to participate in the study |
| **Written informed consent for** | 1) Participation of our study  2) The use of intrathecal morphine intervention |
| ***Allocation*** | |
| **In operating room** |  |
| **Randomization of anesthesia techniques** | The randomization using sealed, opaque envelopes by attending anesthesiologists  1) Intravenous propofol group  2) Inhalational sevoflurane group |
| **Pain relief procedures** | 1) Intrathecal morphine intervention before the induction of general anesthesia  Regimen: 0.2 mg morphine sulphate mixed to total 0.9% saline 1.2 mL   Injection site: lumbar vertebrae 3 and 4  2) Intravenous patient controlled analgesia (IV-PCA) connected to living donors immediately after surgery  Regimen: 1,000 μg fentanyl and 0.3 mg ramosetron mix to total 0.9% saline 100 mL  Program: 1 mL bolus injection without basal infusion of the IV-PCA solution and with a lockout time of 10 min  3) Rescue IV opioid drugs administration in the post-anesthesia care unit and ward (pain score more than 7 on a numeric rating scale [NRS]) |
| **Balanced anesthesia with multiple vital monitoring during surgery** | |
| ***Follow-up during 24 h postoperatively*** | |
| ***Primary outcomes*** |  |
| **The Korean version quality of recovery-40 questionnaire** | 1) Global score 2) Subscale scores: physical comfort, emotional state, psychological support, physical independence, and pain |
| ***Secondary outcomes*** |  |
| **Successful ambulation rate (%)** | 1) Early ambulation 2) Late ambulation |
| **Ambulation (foot-steps)** | 1) Early ambulation 2) Late ambulation 3) Total ambulation |
| **Peak NRS score on wound site** | 1) at rest 2) at cough |
| **Requirement of IV opioid** | 1) Total amount of IV-PCA infusion (mL) 2) Frequency of rescue IV opioid administration |
| **Complications** | Nausea/vomiting; headache; shivering; respiration depression; pruritus |
| **Laboratory variables** | WBC count (x 10^9^/L); neutrophil (%); lymphocyte (%); hemoglobin (g/dL); platelet count (x 10^9^/L); creatinine (mg/dL); albumin (g/dL); sodium (mEq/L); potassium (mEq/L); chloride (mEq/L) |
| ***Follow-up during postoperative hospital administration*** | |
| **Clavien-Dindo classification** | Degree of surgical complications |
| **Total hospital period (days)** |  |
| ***Analysis of study data*** | |
